# Psychological Resilience in Latin America Nursing Students Using the Wagnild and Young Scale: A Scoping Review

**DOI:** 10.3390/ijerph22091425

**Published:** 2025-09-12

**Authors:** William Donegá Martinez, Marco Antonio Ribeiro Filho, Tiago Casaleiro, Marcos Sanches Rodrigues, Emerson Roberto dos Santos, Daniele Nunes Longhi Aleixo, João Daniel de Souza Menezes, Matheus Querino da Silva, Renato Mendonça Ribeiro, Luiz Vianney Saldanha Cidrão Nunes, Rauer Ferreira Franco, Amanda Oliva Spaziani, Marli de Carvalho Jerico, Alex Bertolazzo Quitério, Weslley dos Santos Borges, Christian Guilherme Capobianco dos Santos, Maysa Alahmar Bianchin, Luís Cesar Fava Spessoto, Maria Helena Pinto, Fernando Nestor Facio Júnior, Ronize Aparecida Domingues de Almeida Prado, Ana Paula Bernardes da Rosa, Marlene da Silva, Sabrina Ramires Sakamoto, Neuza Alves Bonifácio, Suzimar de Fatima Benato Fusco, Rita de Cássia Helú Mendonça Ribeiro, Denise Cristina Mós Vaz Oliani, Antônio Hélio Oliani, Júlio César André

**Affiliations:** 1 Center for Studies and Development of Health Education—CEDES, São José do Rio Preto Medical School—FAMERP, São José do Rio Preto 15090-000, Brazil; marcoribeirofilho@gmail.com (M.A.R.F.); marcos.rodrigues@edu.famerp.br (M.S.R.); emerson.santos@edu.famerp.br (E.R.d.S.); daniele.aleixo@edu.famerp.br (D.N.L.A.); joao.menezes@edu.famerp.br (J.D.d.S.M.); lvcidrao@hotmail.com (L.V.S.C.N.); marli@famerp.br (M.d.C.J.); alex.quiterio@edu.famerp.br (A.B.Q.); maysa@famerp.br (M.A.B.); fernando.nestor@famerp.br (F.N.F.J.); julio.andre@edu.famerp.br (J.C.A.); 2 Grupo Autónoma-Escola Superior de Enfermagem São Francisco das Misericórdias, R. Gen. Firmino Miguel 6, Greenpark, 1600-300 Lisbon, Portugal; tcasaleiro@esesfm.pt; 3 São José do Rio Preto Medical School—FAMERP, São José do Rio Preto 15090-000, Brazil; matheusquirino@hotmail.com (M.Q.d.S.); christian.santos@edu.famerp.br (C.G.C.d.S.); mariahelena@famerp.br (M.H.P.); ritadecassia@famerp.br (R.d.C.H.M.R.); 4 Ribeirão Preto School of Nursing, USP, Ribeirão Preto 14040-902, Brazil; drrenatoribeiroenf@gmail.com; 5 Universidade Brasil, Fernandópolis 15600-000, Brazil; rauerf@hotmail.com (R.F.F.); spazianimedicina@gmail.com (A.O.S.); weslley.borges@ub.edu.br (W.d.S.B.); 6 Votuporanga City Hall Municipal Health Department, Votuporanga 15503-452, Brazil; divisao.assistencial@votuporanga.sp.gov.br; 7 UNORTE—University Centre of Northern São Paulo, São José do Rio Preto 15020-040, Brazil; paulabernardes@unorte.edu.br; 8 Escola EMEF Coronel José Pedro da Motta, Catanduva 15813-110, Brazil; pmottadirecao@catanduva.sp.gov.br; 9 Penápolis Faculty of Philosophy, Sciences and Letters, Penápolis Educational Foundation, Penápolis 16303-068, Brazil; ramiressabrina@hotmail.com; 10 Institute of Health Sciences, Paulista University, Araçatuba 16018-555, Brazil; neuzabonifacio@gmail.com; 11 Faculty of Veterinary Medicine, Universidade Estadual Paulista Júlio de Mesquita Filho, Araçatuba 16050-680, Brazil; 12 School of Nursing—UNICAMP, Campinas 13083-887, Brazil; sbenato@unicamp.br; 13 University Hospital Center Cova da Beira, University of Beira Interior, 6200-251 Covilhã, Portugal; vaz.oliani@gmail.com (D.C.M.V.O.); oliani@famerp.br (A.H.O.)

**Keywords:** psychological resilience, nursing students, Wagnild and Young scale, academic stress, mental health, scoping review

## Abstract

Nursing students frequently experience considerable stress, impacting their mental well-being and preparedness for professional practice. Psychological resilience is paramount in navigating these demands. This scoping review synthesized studies on resilience levels in nursing students, particularly those in their entry year, utilizing the Wagnild and Young Resilience Scale within the Latin American academic context. Following JBI methodology and PRISMA guidelines, a systematic search identified six relevant studies. Findings indicate that newly enrolled nursing students often exhibit low to moderate resilience, which may decline during the first academic year. Importantly, resilience acts as a protective factor against psychoemotional stress, depressive symptoms, and poor sleep quality. Family support, engagement in leisure activities, and course satisfaction positively correlate with higher resilience. These findings underscore the imperative for educational institutions to actively integrate resilience-building strategies, such as curricular adjustments and psychoeducational programs, to bolster student well-being and cultivate competent future professionals. Further longitudinal research is essential to deepen understanding and evaluate intervention efficacy.

## 1. Introduction

Psychological resilience is defined as the human capacity to adapt and recover from trauma, adversity, difficulties, and daily life stressors [1,2,3,4]. This intricate psychological construct involves emotional and cognitive processes that enable individuals to maintain or restore mental equilibrium in the face of challenging circumstances [1,2,3,4]. Nursing students experience anxiety and stress during their university years, particularly when initiating clinical placements that involve direct patient care. These clinical practices frequently result in difficulties, physical exhaustion, and mental health impacts due to first experiences in hospital environments, which can significantly compromise the quality of care and patient safety [2,5,6].

Contemporary educational discourse increasingly emphasizes resilience as a protective factor against academic stress-related adverse outcomes, while simultaneously serving as a preparatory foundation for the challenging professional healthcare environment that awaits graduates. Evidence suggests that numerous students demonstrate limited stress tolerance capacity, frequently experiencing health deterioration throughout their academic trajectory. Emerging scholarly investigations underscore the importance of fostering resilience competencies among both nursing students and practicing professionals to strengthen adaptive responses when confronting patient-related distressing circumstances [3,6,7,8,9].

The healthcare sector is characterized by inherent stressors that may precipitate psychological distress and anxiety responses. Professional demands, inadequate organizational resources, restricted professional autonomy, exposure to patient pain and suffering, patient mortality experiences, and additional occupational stressors can contribute to psychological morbidity among susceptible healthcare workers. Consequently, it becomes important that students, as emerging healthcare professionals, enter their careers with enhanced psychological fortification rather than compromised resilience capacity [8,9,10].

The impact of nursing education demands on students’ physical and mental health is a topic of growing concern. Various studies corroborate this by emphasizing the inclusion of self-care and resilience education in nursing curricula, recognizing the importance of preparing future professionals to deal with the physical and emotional challenges of the profession [5,11].

Academic demands beyond the formal curriculum, including exposure to human suffering and the weighty responsibility of life-and-death decision-making, may exceed students’ coping mechanisms, thereby inducing significant psychological strain. Non-academic stressors, including emotional frustration, academic disengagement, economic constraints, geographical separation from family support systems for educational purposes, and reliance on complex public transportation networks, constitute persistent challenges within the university experience. Furthermore, developmental transitions encompassing adult role assumption, interpersonal relationship navigation, socioeconomic burden management, and career market preparation emerge as prominent stressors [12,13].

Individual resilience capacity demonstrates considerable variability, influenced by intrinsic personality traits and the broader sociocultural environment within which individuals function. Those possessing enhanced resilience demonstrate superior adaptability to contemporary employment challenges and obstacle navigation, as they exhibit greater facility in managing daily adversities and recovering from setbacks [14,15,16].

Students enrolled in higher education programs and healthcare professionals, such as those in nursing, pharmacy, social work, and medicine, face a variety of stressors. These factors include academic pressures, intense workload, development of professional competencies, social integration into the profession, non-explicit aspects of the curriculum, entry into clinical practice, and establishment of interpersonal relationships with colleagues and patients [14,15,16,17,18,19].

This stress can have adverse effects on the physical and psychological well-being of students and healthcare professionals, and consequently negatively impact the quality of care provided to patients.

Healthcare professions characterized by intensive interpersonal engagement, including medicine, psychology, nursing, and physiotherapy, demonstrate heightened susceptibility to stress-related complications, given the intrinsic occupational stress [14,15,16,17,18,19].

Self-care practices constitute essential elements in resilience enhancement processes. Nevertheless, such development requires individuals to maintain conscious awareness of their well-being status and achieve comprehensive biopsychosocial equilibrium. Within higher education contexts, pedagogical frameworks must incorporate student competency development in stress management strategies throughout their academic progression. Resilience represents an important academic success component, with recreational engagement, course satisfaction, and familial harmony serving as contributory factors to healthcare professional resilience [14,15,20].

Educational faculty and institutional healthcare organizations serve as important partners in promoting healthy lifestyle adoption during university years through targeted educational interventions. Resilience-strengthening health initiatives could be systematically developed and integrated within academic environments. Robust resilience cultivation proves essential for student adaptation within nursing educational settings and subsequent professional practice [4,12,21,22,23].

Nursing students, alongside other healthcare discipline graduates, experience vulnerability through the tension between rational and emotional responses when confronting human frailty, potentially precipitating sadness, pessimism, distress, apprehension, uncertainty, anxiety, and inadequacy feelings. Moreover, heightened responsibility perceptions, socioeconomic challenges, bereavement experiences, and academic pressures constitute risk factors for psychological distress and mental health disorders [18,24,25,26].

The Wagnild and Young Resilience scale (WY-RS) is a widely recognized and utilized psychometric instrument designed to measure an individual’s level of psychological resilience. Developed by Wagnild and Young in 1993, this scale provides a quantitative assessment of an individual’s ability to cope with stress and adversity, offering insights into core resilience characteristics such as purpose, self-reliance, resourcefulness, perseverance, and meaningfulness [27,28]. Its extensive use in diverse populations and clinical settings, including healthcare education, stems from its established psychometric properties and its capacity to identify nuanced aspects of resilience. For the scope of this review, the WY-RS was chosen as a specific criterion for study inclusion to ensure a consistent and comparable measure of resilience across the synthesized literature. This focus allows for a more targeted and in-depth understanding of resilience levels and associated factors within nursing students, particularly those in Latin American contexts, as measured by a standardized and validated tool.

Despite the growing body of literature on student well-being and resilience, there remains a need for a comprehensive synthesis focused on specific populations and contexts [29].

Nevertheless, the limited availability of empirical research examining nursing student resilience, the imperative to prepare nursing students with adequate resilience for strengthened rather than compromised workforce entry, and the important role of actualizing self-care and resilience development as predictive indicators of academic and professional performance provide compelling justification for this investigation [29]. Therefore, this scoping review aims to comprehensively map and synthesize existing evidence on the psychological resilience levels of nursing students, particularly those in their entry year, utilizing the Wagnild and Young Resilience scale, within the academic context of Latin America. This approach will allow us to identify key concepts, illuminate prevailing trends, and pinpoint knowledge gaps, thereby providing a foundation for future research and evidence-based interventions in nursing education.

## 2. Materials and Methods

### 2.1. Study Design

This is a qualitative scoping review study, a type of bibliographic survey conducted according to the methodology defined by the JBI Manual for Evidence Synthesis (2020) [30], whose method aims to map key concepts, clarify and identify knowledge gaps, following these stages: (1) formulation of the research question and definition of search descriptors; (2) literature search in international databases; (3) reading titles and abstracts of articles for selection according to inclusion and exclusion criteria; (4) full reading of selected studies and data mapping; (5) summarization and critical analysis of results; (6) presentation of main results following the proposed reference framework [30]. The protocol was developed and registered on the Open Science Framework (OSF) platform under DOI https://doi.org/10.17605/OSF.IO/52RA3 (registered on 24 September 2022; accessed on 24 September 2022) to ensure review transparency and was published as an article in the scientific journal Research, Society and Development under DOI https://doi.org/10.33448/rsd-v12i7.42624.

### 2.2. Guiding Question and Inclusion Criteria

The guiding question for this scoping review was meticulously delineated using the PCC acronym (P = Population, C = Concept, and C = Context), which served as a compass for defining study inclusion criteria. The central question formulated was:

What are the resilience levels of nursing course entrants using the Wagnild and Young Resilience scale, and what are the associated factors and implications of these findings?

#### 2.2.1. Population

In this study, the primary focus population comprises nursing students entering higher education. The choice of this specific cohort is justified by the need to deepen knowledge about resilience at a crucial moment in academic training, considering that there are few detailed studies about their levels using the Wagnild and Young Resilience scale [31]. However, to provide a comprehensive understanding of the topic and contextualize findings, the review also included studies addressing the broader sociodemographic profile of nursing students and, to a lesser extent, other health areas, whose results may offer insights into factors associated with resilience and academic training dynamics [15].

#### 2.2.2. Concept

This scoping review considered studies that explored resilience from various perspectives, going beyond simple measurement. The central concept encompassed not only the description of nursing students’ resilience levels, as measured by the Wagnild and Young Resilience scale, but also extended to the analysis of a broader set of elements. These include:

- Sociodemographic and academic factors that may be associated with or influence student resilience.
- Resilience impacts on student well-being, mental health, and academic performance.
- Methodological characteristics of studies, including considerations about the validity and reliability of the Wagnild and Young Resilience scale, as well as its limitations.
- Implications and recommendations derived from findings for professional practice, nursing education, and future research agenda.

The objective was to compile and synthesize all information related to these students’ resilience, beyond mere quantification, aiming for a deep and multifaceted understanding of the phenomenon.

#### 2.2.3. Context

The review context initially sought to encompass global literature without geographical restrictions, aiming to identify all existing sources of evidence on the topic. However, analysis of included studies revealed a predominant concentration of research in Latin America, specifically in Brazil and Peru. This geographical particularity of the studies found shaped the review context, allowing for an in-depth analysis of academic and health realities in these regions (Latin America), and highlighting the majority contribution of these countries to the body of evidence on the topic, even though the initial scope was broader. This regional concentration reflects the available evidence base rather than a deliberate limitation of the search strategy, allowing for a focused and in-depth understanding of resilience within these specific Latin American educational contexts. While this may limit direct generalizability to the entire continent, it provides valuable insights into the prevalent patterns and influencing factors within the identified regions.

#### 2.2.4. Procedures

This research followed the recommended stages for conducting a scoping review, according to the PRISMA-ScR (Preferred Reporting Items for Systematic Reviews and Meta-Analyses extension for Scoping Reviews) guidelines, 2020 [32], which are: identification of the research question; search for relevant studies; selection of material to be reviewed; data extraction and analysis; preparation and presentation of the review; submission of the text to peers for analysis. The search for studies in consulted databases was operationalized by the first author and their decisions were validated by a second researcher, independently. Both evaluators have expertise in scoping reviews. The research was developed from April 2022 to July 2024. The literature search and data collection were concluded by late 2023, encompassing publications up to that point. The period from January to July 2024 was dedicated to data analysis, synthesis, and manuscript preparation, ensuring that the review incorporates the most recent relevant literature available within the specified search timeframe.

### 2.3. Search Strategy and Database Selection

The search strategy and database selection were defined by a professional specialized in digital search strategy in conjunction with the authors.

The search was conducted in the following databases: CINAHL, PubMed, SCOPUS, Web of Science, and LILACS. The search strategy was developed with the assistance of a specialized librarian and included MeSH terms, DeCS, and keywords related to ‘nursing students’, ‘psychological resilience’, and ‘Wagnild and Young scale’. The complete strategy for PubMed was: ((“Nursing Students” [Mesh] OR “nursing student*” [tiab]) AND (“Resilience, Psychological” [Mesh] OR resilien* [tiab]) AND (“Wagnild and Young” [tiab] OR “resilience scale” [tiab])). This strategy was adapted for other databases. Searches were limited to publications from the last 7 years (i.e., from 2017 to 2023). This timeframe was chosen to capture the most recent and relevant literature reflecting contemporary academic and professional contexts in nursing education at the time the search was concluded. The search included English, Portuguese, and Spanish languages. Grey literature, specifically theses and dissertations, was searched in Google Scholar and thesis and dissertation repositories to identify potential studies and to gain a comprehensive overview of the research landscape. Searches were conducted on 20 December 2023. Mendeley software [33] was used to manage references and remove duplicates.

Search strategies for all databases searched can be seen in Supplementary S1.

The scientific databases used in the searches confer a degree of credibility and quality fundamental to any scientific work, allowing researchers to base themselves on content appropriate to their practice to be developed.

By using a scientific database for such a task, it is ensured that the articles present there have undergone evaluation by at least 2 (two) reviewers (professionals in the area in question to the theme, with relevant experience), in addition to factors such as originality, relevance, scientific writing, among other parameters generally required for scientific work.

The non-use of other databases does not mean they do not correspond to the aspects listed above, but rather to the fact that those used provide more results in the searches conducted.

### 2.4. Eligibility Criteria

Publications in English, Spanish, and Portuguese languages from the last 7 years (2017 to 2023), available in the following databases: Cumulative Index to Nursing and Allied Health Literature (CINAHL); National Library of Medicine (PubMed); SCOPUS; WEB OF SCIENCE; MEDLINE, EMBASE, Scientific Electronic Library Online (SCIELO) and Google Scholar. Articles available in full; original articles, with quantitative and qualitative approaches, primary studies, systematic reviews, meta-analyses and/or meta-syntheses, books and guidelines, published in indexed sources, that answer the established question.

### 2.5. Exclusion Criteria

Excluded from this study were incomplete articles, duplicate documents, opinion publications, consensus documents, retractions, editorials, websites and advertisements published in media, event abstracts, event proceedings, printed and online books, documents in press, grey literature, documentary videos and films. However, doctoral and master’s theses and dissertations were considered eligible for inclusion despite being classified as grey literature, provided they met the specific criteria for relevance to the research question and demonstrated the necessary methodological rigor for analysis.

The study selection process was conducted in two main stages:

1. Title and abstract screening: Two independent reviewers (W.D.M. and T.C.) evaluated the titles and abstracts of all records identified in the searches. Studies that potentially met the inclusion criteria were selected for full-text reading.
2. Full-text evaluation: The same two reviewers independently evaluated the full texts of studies selected in the first stage. Disagreements in both stages were resolved through discussion between reviewers, with the participation of a third reviewer (J.C.A) when necessary.

Before beginning the formal selection process, a pilot was conducted with 50 records to calibrate reviewers and refine selection criteria. Rayyan software (Rayyan Systems, Inc., Cambridge, MA, USA) [34] was used to manage the selection process.

**Figure 1:**
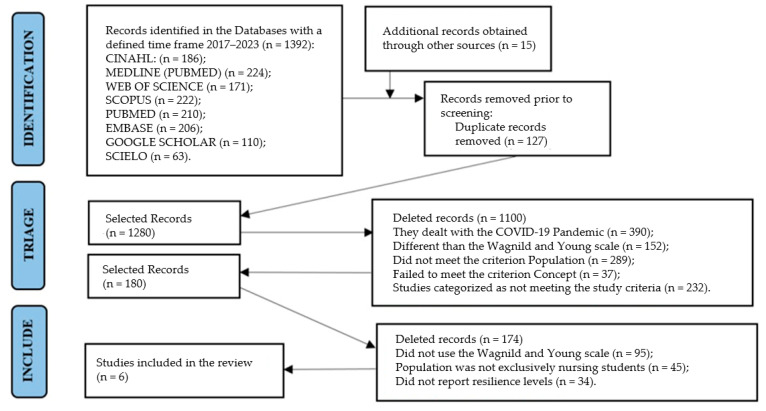
PRISMA-ScR flowchart of study selection process. (2025; Source: adapted from [35]).

The study selection process is detailed in the PRISMA-ScR flowchart (Figure 1). Initially, we identified 1392 records through database searches and 15 additional records through other sources. After removing 127 duplicates, 1280 records were screened by title and abstract. Of these, 1100 were excluded for not meeting inclusion criteria. We evaluated 180 full-text articles, of which 174 were excluded for the following reasons: did not use the Wagnild and Young scale (n = 95), population was not exclusively nursing students (n = 45), did not report resilience levels (n = 34). Finally, 6 studies were included in the qualitative synthesis of the scoping review.

List of included studies and their characteristics is available in Supplementary S2.

### 2.6. Data Extraction

A standardized data extraction form was developed specifically for this review, based on study objectives and previous literature on resilience in nursing students. The form was created in REDCap [36,37,38] and included the following main sections:

1. Study information: authors, year of publication, country, study design
2. Sample characteristics: size, mean age, gender distribution, course year
3. Methodology: sampling method, inclusion/exclusion criteria
4. Wagnild and Young scale details: version used, application method
5. Resilience results: mean scores, standard deviation, level categorization
6. Factors associated with resilience: sociodemographic, academic, psychological variables
7. Interventions (if applicable): type, duration, results

The form was piloted with five randomly selected studies and refined based on this experience. Additional fields for qualitative notes were included to capture important contextual information. The final form used is available in Supplementary S3.

### 2.7. Data Analysis and Synthesis

The analysis and synthesis of extracted data followed a narrative synthesis approach, suitable for integrating results from studies with diverse methodologies [39,40,41]. The process involved the following stages:

1. Initial organization: Extracted data were organized in tables, separating information about study characteristics, methodologies, and results [42,43].
2. Descriptive analysis: A descriptive analysis of included study characteristics was performed, such as geographical distribution, study designs, and sample sizes [44,45].
3. Thematic analysis: Qualitative data were subjected to thematic analysis to identify patterns and recurring themes related to resilience in nursing students [46,47].
4. Quantitative synthesis: For quantitative data on resilience levels, weighted means and confidence intervals were calculated when possible. Heterogeneity between studies was evaluated and discussed [48,49].
5. Integration: Results from qualitative and quantitative analyses were integrated to provide a comprehensive view of the current state of knowledge about resilience in nursing students [50,51].

The analysis was conducted independently by two reviewers (D.N.L.A. and J.D.d.S.M.), using NVivo 12 software for thematic analysis [52,53]. Discrepancies were resolved through discussion and consensus [54,55]. The final synthesis was reviewed by all authors to ensure its accuracy and comprehensiveness.

## 3. Results

This section describes and synthesizes the main findings of the included studies that address resilience levels in nursing students, with special focus on first-year students, and the utilization of the Wagnild and Young Resilience scale. The information is organized according to the general characteristics of the studies, methodological aspects, observed resilience levels, associated factors, and conclusions and implications. Supplementary S4 compiles the tables summarizing the results. This synthesis, while based on a limited number of studies (n = 6), provides valuable insights into the specific context of nursing students’ resilience in Latin America, particularly those in their entry year, measured by the Wagnild and Young Resilience scale.

To provide a clear and concise overview of the primary studies identified and included in this scoping review, their essential characteristics are systematically detailed in Table 1, specifying titles, publication types, countries of origin, publication years, and the databases contributing to their identification through our comprehensive search strategy.

### 3.1. General Characterization of Selected Studies

To address the central question of this review, two studies were identified that directly address the population of nursing program entrants and utilize the Wagnild and Young Resilience scale: the study by Silva (2017) and that by Souza et al. (2020) [12,56]. Additionally, the study by Moraes-Filho et al. (2020), although not exclusively focused on nursing entrants, included nursing students in its sample of health courses, utilizing the same scale and providing a broader context regarding resilience levels in undergraduate health students [15].

The study by Silva (2017), a doctoral dissertation published in Brazil, had as its primary objective to verify significant alterations in health status, resilience levels, and quality of life of undergraduate nursing students after the first academic year, as well as to analyze causal relationships between psychoemotional stress, depressive symptoms, sleep quality, and resilience in explaining quality of life during the inaugural year [56]. Due to its longitudinal nature, it proves particularly relevant for understanding the dynamics of resilience during this critical period.

Complementarily, the research by Souza et al. (2020), a peer-reviewed scientific article also from Brazil, sought to investigate the relationship between stress and resilience among first-year nursing students at two public universities in São Paulo [12]. This cross-sectional study offers a panorama of resilience levels at the beginning of academic training.

Finally, the article by Moraes-Filho et al. (2020), equally Brazilian and peer-reviewed, analyzed the association between sociodemographic and academic factors and resilience levels of undergraduate students in health programs, including nursing [15]. Although its sample is more comprehensive, its results regarding the Wagnild and Young scale contribute to a richer understanding of resilience levels in a university health population.

Regarding the demographic characteristics of samples directly related to nursing entrants, the longitudinal study by Silva (2017) (longitudinal phase) had a mean age of 20.73 years (SD ± 4.4) in March 2016, rising slightly to 20.90 years (SD ± 5.1) in December of the same year [56]. Female predominance was pronounced, with 84.6% in March and 88.0% in December. Similarly, Souza et al. (2020) reported a mean age of 20.73 years (SD = 4.4) and a majority of 84.6% female participants, reflecting the typical profile of the nursing student population [12]. In both studies, participants were first-year students or first and second semester students, aligning perfectly with the focus on “entrants”. It is notable that both Silva (2017) and Souza et al. (2020) reported identical mean age and standard deviation (20.73 years, SD = 4.4) and female participant percentages (84.6%) for their respective samples, indicating a consistent demographic profile among first-year nursing students in the Brazilian context during the study periods [12,56].

### 3.2. Geographic and Temporal Distribution of Studies

The studies included in the review present a geographic concentration in South America, specifically in Brazil and Peru, and a temporal distribution spanning recent years, indicating a recent and continuous interest in the theme of resilience in nursing students.

The majority of studies originate from two countries:

- Brazil: Three studies were developed in Brazil: Silva (2017), Moraes-Filho et al. (2020), and Souza et al. (2020) [12,15,56]. These studies focused primarily on institutions located in the states of São Paulo and the Federal District (Brasília).
- Peru: Three studies were conducted in Peru: Mejía (2019), Salvador (2023), and Yesenia (2023) [57,58,59]. Public and private universities and institutes in Lima and Ica were the settings for these investigations.

This geographic distribution reflects a regional approach to understanding resilience, concentrating on the academic and health realities of Latin American countries.

Data collection for the studies occurred predominantly in recent years, as detailed below:

- The longitudinal study by Silva (2017), although published in 2017, had its data collected in two distinct phases during 2016 (March and December) [56].
- The research by Mejía (2019), from Peru, collected data during the second academic semester of 2018 [57].
- For Moraes-Filho et al. (2020) and Souza et al. (2020), both published in 2020 in Brazil, the exact year of recruitment and data collection is not specified, but it is inferred that collections occurred in the immediately preceding years or in the same year of publication, probably between 2019 and 2020 [12,15].
- The most recent studies by Salvador (2023) and Yesenia (2023), both from Peru and published in 2023, do not detail the precise year of data collection, but it is reasonable to assume that the research was conducted in the year of publication or in the most recent years, that is, between 2022 and 2023 [58,59].

This concentration of publications and data collections from 2016 onwards, with peaks in 2019, 2020, and 2023, demonstrates growing interest and research activity regarding resilience in nursing students in Latin America in recent years.

### 3.3. Methodological Characteristics for Resilience Assessment

All studies of interest employed the Wagnild and Young Resilience scale (WY-RS) as the primary instrument for measuring resilience. The choice of this scale is a crucial point, as it ensures comparability of results and relevance to the review question.

In the study by Silva (2017), the research adopted a quantitative approach with a longitudinal design [56]. The Wagnild and Young scale was utilized in conjunction with other instruments such as the Assessment of Stress in Nursing Students Instrument (ASNS), the Center for Epidemiologic Studies Depression Scale (CES-D), the Pittsburgh Sleep Quality Index (PSQI), and the World Health Organization Quality of Life Instrument—Abbreviated Version (WHOQOL-Bref). Data analysis involved ANOVA for mixed models and Structural Equation Modeling (SEM), a robust methodology for investigating complex relationships. The scale calibration phase, mentioned by the study, emphasizes concern with instrument validity, although the authors pointed out the need for scale respecification to a unifactorial model due to suboptimal fit indices [56].

The study by Souza et al. (2020) employed a cross-sectional and quantitative design [12]. In addition to the Wagnild and Young Resilience scale, a sociodemographic and academic questionnaire and the Assessment of Stress in Nursing Students Instrument (ASNS) were utilized. Data analysis included descriptive statistics, Cronbach’s alpha coefficient for reliability, and Pearson’s correlation test, with a significance level of *p* < 0.05. It is important to note that this study did not explore the multifactorial structure of the scale, focusing on global scores or by domains.

In turn, Moraes-Filho et al. (2020) conducted a cross-sectional and analytical study, also with a quantitative approach, utilizing the Wagnild and Young Resilience scale (adapted to the Brazilian context) and a sociodemographic and academic questionnaire [15]. Data analysis was processed in SPSS v16.0, with descriptive statistics, chi-square tests, and a significance level of *p* < 0.05. This study provided detailed data on the means of the scale factors.

The homogeneity in the utilization of the Wagnild and Young scale among these studies, despite variations in design and statistical analyses, strengthens the foundation for synthesizing findings regarding resilience levels in this specific population.

### 3.4. Resilience Levels of Nursing Program Entrants

The findings regarding resilience levels of nursing program entrants, measured by the Wagnild and Young Scale, present important nuances among studies, indicating the complexity of resilience assessment and classification.

In the longitudinal study by Silva (2017), which followed nursing students from their first year, the overall mean resilience score was 120.36 in March (beginning of the period) and 119.86 in December (end of the period), with no statistically significant change over this time [56]. The interpretation of these values is crucial: participants were situated at the threshold between “reduced resilience” (below 121) and “moderate resilience” (between 121 and 145). Notably, at the end of the academic year, there was an inclination toward “reduced resilience” [56]. Although the general means of the scale factors (Action and Values, Independence and Determination, Self-confidence and Adaptability) also showed no significant changes, the values in December 2016 were: Action and Values: 4.97; Independence and Determination: 4.00; Self-confidence and Adaptability: 5.23. These data suggest that, even without a statistically significant decline, resilience levels of entrants may tend toward a state of greater vulnerability throughout the first academic year.

On the other hand, the research by Souza et al. (2020), focused on the first years of nursing, revealed that 51% of students presented low resilience and 45% moderate resilience, totaling 96% of the sample in these two categories [12]. The level of “high resilience” was not explicitly reported, suggesting that a very small portion, or none, of the sample fell into this classification. The “Actions and Values” domain was the one that most contributed to resilience in the sample, with a mean of 5.06 (SD = 0.84) [12]. These results reinforce the perception that a considerable portion of entrants may present resilience levels that demand attention, especially considering the challenges of the academic environment.

In contrast to the more focused findings on entrants, the study by Moraes-Filho et al. (2020), which included a broader sample of undergraduate health students (including nursing, physiotherapy, and pharmacy), presented a slightly different and, in some ways, more optimistic resilience distribution [15]. In this group, 6.1% of students had low resilience, 71.7% presented moderate resilience, and 21.7% demonstrated high resilience [15]. The means of the scale factors were: Actions and Values: 5.88 (SD = 0.44); Independence and Determination: 4.97 (SD = 0.20); Self-confidence and Adaptability: 5.87 (SD = 0.77). Although this sample is not exclusive to nursing entrants, the higher percentage of moderate resilience and the presence of high resilience in this more heterogeneous group may indicate that, in broader populations of health students, levels may be somewhat higher, or that other factors (such as course phase) may influence results.

The joint analysis of these studies suggests that, for nursing entrants specifically, resilience levels tend to be lower or at the threshold between reduced and moderate, as indicated by Silva (2017) and Souza et al. (2020) [12,56]. The comparison with Moraes-Filho et al. (2020), which encompasses a broader spectrum of health students, raises the hypothesis that the specificity of the nursing course in the first year or the longitudinal nature of adaptation may influence these resilience levels [15].

### 3.5. Associated Factors and Impacts of Resilience

Resilience does not exist in isolation; it interacts with various sociodemographic and academic factors, and its levels can have significant impacts on the well-being and mental health of nursing students. The analyzed studies provide valuable insights into these interactions.

Silva (2017) evidenced that resilience acts as a crucial protective factor in relation to psychoemotional stress (β = −0.51) and depressive symptoms (β = −2.75), in addition to improving sleep quality (β = −0.17) [56]. This finding is fundamental, as it suggests that, even if entrants’ resilience levels are not elevated, any degree of resilience already plays a mitigating role against academic environment adversities. The author emphasizes that resilience allows students to perceive stressors as challenges and adopt adaptive behaviors, contributing to better academic performance and general health status. However, in this study, resilience did not demonstrate a direct effect on students’ general quality of life [56].

Despite not finding statistically significant correlations between stress and resilience, Souza et al. (2020), in their discussion, emphasize that resilience can act as a protective factor against academic stress, anxiety, depression, and burnout [12]. This observation, even without statistical confirmation in the study itself, reflects the general literature on the theme and reinforces the theoretical importance of resilience in this context.

The study by Moraes-Filho et al. (2020), although with a broader population, brought to light significant associations between resilience levels and housing arrangement (*p* = 0.003), engagement in leisure activities (*p* = 0.010), and satisfaction with the academic program (*p* = 0.001) [15]. It was observed that resilience was positively correlated with residing with family, participating in leisure activities, and being satisfied with the course. These associations suggest that family support, the ability to engage in pleasurable activities, and positive perception of academic training are elements that can strengthen resilience in health area students [15].

In summary, the results point to resilience as a shield against stress and depressive symptoms, with potential associations with environmental factors and academic satisfaction. The absence of direct correlation between stress and resilience in some studies may indicate a more complex relationship or the need for longitudinal approaches to capture the dynamics.

## 4. Discussion

This scoping review had as its primary objective to investigate resilience levels of nursing program entrants, utilizing the Wagnild and Young Resilience Scale. The findings of the selected studies offer a multifaceted understanding of these students’ profiles, the dynamics of resilience during academic training, and the practical and theoretical implications for the nursing field.

### 4.1. Sociodemographic Profile and Its Impacts on Academic Trajectory

The analyzed studies consistently point to a predominance of female participants in the nursing student sample [12,15,56,57,58,59]. This demographic pattern aligns with established trends in nursing education, though the underlying factors contributing to this distribution warrant further investigation [12,60]. Although nursing has expanded to all genders, this persistence of female predominance continues to shape the educational environment.

Regarding marital status and the presence of children, it is observed that the majority of students do not have a partner or children, although some studies show the existence of students with children [12,15,56]. The absence of significant family responsibilities may positively influence academic dedication and performance by reducing external demands and stress, allowing greater time and focus on studies [12,15]. This scenario, combined with financial dependence on family resources for many students, configures a context in which time management and the ability to deal with academic stress become crucial for success [12,15,56]. Prolonged exposure to stress, regardless of family responsibilities, can diminish individual resilience, affecting emotional balance and the brain’s adaptive capacity [59].

### 4.2. Dynamics of Resilience Levels and Associated Factors

The general findings indicate that nursing students, especially entrants, tend to present resilience levels that vary from moderate to high, with an inclination toward moderate or even reduced resilience in some contexts, as observed in the study by Silva (2017), which pointed to a tendency toward “reduced resilience” at the end of the first academic year [56]. The differences in resilience levels observed between studies in Brazil and Peru suggest that contextual factors such as academic environment, institutional support, and the socioeconomic and cultural particularities of each country can significantly influence the development of this characteristic [12,15,57,59].

Resilience stands out as a fundamental protective factor against the deleterious effects of psychoemotional stress and depressive symptoms, in addition to contributing to better sleep quality [56]. It enables students to perceive stressors as challenges to be overcome, rather than insurmountable threats, promoting adaptive behaviors and better general well-being [56]. Although not all studies found statistically significant correlations between stress and resilience, the literature reinforces the role of resilience as a buffer against academic stress, anxiety, depression, and burnout [12].

Additionally, factors such as housing arrangement (residing with family), engagement in leisure activities, and satisfaction with the academic program were positively correlated with higher resilience levels [15]. This suggests that social support, the ability to decompress, and a positive perception of the educational experience are essential elements that can strengthen students’ capacity to deal with the demands of the nursing course.

### 4.3. Implications for Practice and Teaching in Nursing

The findings from this scoping review suggest that the promotion of resilience emerges as a potentially important necessity for nursing education, though further research is needed to establish definitive causal relationships. The nursing education environment, characterized by high demand and situations of stress, suffering, and adversities, requires that future professionals develop a robust adaptive capacity [14,15,16,17,18,19,20].

Based on the limited evidence available, higher education institutions may consider reviewing and adjusting their curricula to reduce academic overload, incorporating alternative assessment methods and considering the effects of stress on programmatic structure [12,20,56]. The evidence suggests that promotion of self-efficacy, offering students greater control and choices over their educational trajectory, may strengthen their capacity to deal with academic and professional pressures [15].

While the current evidence base is limited, it appears fundamental that educators focus not only on developing individual skills, such as emotional intelligence and mindfulness, but also on adapting the social and organizational contexts in which students are inserted [15]. Practical recommendations, requiring further validation through rigorous research, include:

- Psychoeducational Programs: Consideration of implementation of initiatives focused on developing preventive skills and promoting mental health, expanding the reach of psychological support [58,59].
- Regular Psychological Assessments: Exploration of monitoring students’ mental health from the beginning to the end of the course, for early detection of problems and targeted interventions [58].
- Community Involvement: Investigation of extension of resilience promotion actions beyond students, including staff and even parents, in order to build a more solid support network [57].
- Creation of Healthy Environments: Evaluation of curricular review, provision of social and relaxation spaces, and availability of preventive health services by multiprofessional teams [12,56]. This may include equipped rest areas, fostering physical and mental well-being.

The available evidence suggests that resilience, being a developable rather than innate characteristic, may be cultivated through specific educational strategies, such as teaching problem-solving skills, emotional intelligence, developing a sense of personal control, and creating social support networks [1,12,15,56,57,58,59]. However, the effectiveness of these approaches in nursing education contexts requires further empirical validation. The continuous promotion of resilience potentially benefits academic success and students’ quality of life and may prepare them to be more competent and emotionally balanced professionals, capable of providing quality care to patients [12,15,20,56,57,58,59].

### 4.4. Research Gaps and Recommendations for Future Studies

The critical analysis of the studies revealed important gaps and limitations that should be addressed in future research. The lack of robust longitudinal studies with nursing students, for example, hinders understanding of resilience dynamics over time and comparison of findings at different phases of training [56]. Additionally, the validity and reliability of instruments, such as the Wagnild and Young Resilience Scale, require more in-depth analyses with larger samples, especially in different cultural contexts [31,56].

#### Recommendations for Future Studies Include

- Longitudinal Designs: Following students for more extensive periods (more than one year) to identify significant changes in resilience, quality of life, and sleep quality [56].
- Diversified and Larger Samples: Inclusion of students from different university realities, geographic regions, and socioeconomic contexts to increase generalization of results [12,57,58].
- Investigation of Mediating Factors: Exploration of elements that influence the relationship between resilience and depressive symptoms, as well as other personal, family, and community factors that may impact resilience [57,58,59].
- Intervention Evaluation: Conducting large-scale, high-quality empirical research to test the effectiveness of resilience promotion programs in different educational contexts, utilizing validated and reliable tools for stress management [12,15,20,56,59].
- Factor Analysis and Scale Validation: Deepening the study of the internal structure of the Wagnild and Young Resilience Scale in Brazilian and other populations, aiming to improve its indices and validate constructs [31,56].

These approaches will allow not only greater depth in understanding resilience but will also provide fundamental data for developing more effective interventions, contributing to the sustainability and improvement of daily practices of future nursing professionals [15].

### 4.5. Quality Assessment and Study Limitations

Quality assessment and identification of limitations are essential steps for interpreting results with parsimony and directing future investigations [12,15,56,57,58,59]. The authors of the included studies acknowledged their own restrictions, which is an indicator of scientific rigor.

Silva (2017) pointed out several important limitations [56]. One was the scarcity of detailed descriptions regarding procedures for construct validation and reliability analyses of the utilized instruments. The need to respecify the Wagnild and Young Resilience Scale from a multifactorial to a unifactorial model due to suboptimal fit indices was another critical point, suggesting the need for revision of the scale’s internal structure with larger samples. Additionally, the author questioned whether the one-year follow-up period would have been sufficient to identify substantial changes in sleep quality, resilience, and quality of life, which is a relevant consideration for longitudinal studies of adaptation phenomena. The rarity of longitudinal studies involving nursing students also hindered comparative analysis of her findings [56].

The study by Souza et al. (2020) mentioned as a limitation the restricted availability of studies on resilience in nursing students in Brazil, which may hinder comparisons and deepening of analyses [12]. They also highlighted that the participants’ context was limited to large public institutions in metropolitan areas, which may affect the generalization of results to other universities or geographic realities. Although the statistical methods (Cronbach’s alpha, Pearson correlation) were considered appropriate, limitations related to sample representativeness and the cross-sectional nature of the study were acknowledged [12].

Although Moraes-Filho et al. (2020) did not explicitly state limitations in the available excerpt, the cross-sectional nature of their study implies that it is not possible to establish causal relationships, only associations, a point that is a common limitation in designs of this nature [15]. The diversity of the sample, which included different health area courses, although offering a broader perspective, may, on the other hand, dilute the specificity of findings for nursing entrants.

In summary, the identification of these limitations underlines the complexity of resilience research and the need for future studies with more robust designs, representative samples, and in-depth instrument validations.

### 4.6. Conclusions and Implications for Resilience

The conclusions of the analyzed studies reinforce the importance of resilience in the context of nursing education and point to significant implications for practice, education, and research.

Silva (2017) concluded that, although the employed instruments demonstrated satisfactory validity and reliability, nursing entrants experienced a significant increase in psychoemotional stress, reduction in subjective sleep quality, and intensification of depressive symptoms after the first academic year, without, however, a significant change in general resilience [56]. Resilience, however, was confirmed as a crucial mitigator of psychoemotional stress, a factor that improves sleep quality and decreases the intensity of depressive symptoms. The author emphasized that the nursing education environment possesses the inherent potential to induce illness and suffering, which can impact students’ quality of life. Thus, psychoemotional stress was identified as a strong predictor of health changes, while resilience emerged as a protective factor [56].

The implications for nursing education are clear: Silva (2017) urged institutions to reevaluate their curricula to alleviate academic overload and address challenges in time management and professional communication [56]. She suggests fostering resilience and creating environments that promote student health, including regular psychological assessments for early detection of disorders. For practice, the research underlines the importance of training healthier professionals prepared for job market demands. In terms of research, there is a continuing need for more longitudinal studies and in-depth investigations of resilience in health contexts. Practical recommendations include implementing programs to promote resilience, the feasibility of multidisciplinary support spaces, and even creating rest areas in institutions for student well-being [56].

Souza et al. (2020) concluded that, although moderate stress levels predominated and time management and theoretical work were the most stressful factors, the majority of students presented low or moderate resilience, without significant correlation between stress and resilience [12]. This reinforces the need for academic institutions to promote healthier educational environments and offer psychological support to protect students’ mental well-being. Their recommendations include implementing institutional programs focused on emotional education, resilience development, and early identification of psychological risks related to stress among nursing students [12].

Moraes-Filho et al. (2020), when analyzing health students in general, concluded that these demonstrated moderate to high levels of resilience, and that factors such as engagement in leisure, course satisfaction, and living with family were associated with greater resilience [15]. The implications of this study suggest that promoting supportive environments, leisure, and academic satisfaction can strengthen student resilience, which is crucial for their quality of life and academic performance [15].

In synthesis, the results of this scoping review, based on studies that utilize the Wagnild and Young Scale, indicate that nursing program entrants in Latin America tend to present resilience levels that vary between low and moderate, with an inclination toward vulnerability during the first year. Resilience establishes itself as a crucial protective factor against stress and depressive symptoms, and its promotion is fundamental for the well-being and academic success of these future professionals.

The nursing education environment, characterized by high demand and situations of stress, suffering, and adversities, requires that future professionals develop a robust adaptive capacity [2,5,6,8,14,15,16,17,18,19,20,26].

## 5. Conclusions 

The analyzed studies consistently demonstrate that nursing students, especially entrants, present resilience levels that vary from moderate to high, although with a tendency toward more modest or even reduced levels throughout the first year of training. This resilience is a crucial psychological resource, as it acts as a significant protective factor against psychoemotional stress, depressive symptoms, and negative impacts on sleep quality, elements prevalent in the challenging academic environment of nursing.

Given the influence of the formal curriculum and academic environment on students’ experience, it becomes imperative that educational institutions review and adjust their pedagogical approaches. Reducing academic overload, implementing alternative assessment methods, restructuring curricula to mitigate stress effects, and creating environments that promote self-efficacy and social support are essential strategies. The proactive promotion of resilience through psychoeducational programs, regular mental health assessments, and fostering coping skills are fundamental for the well-being and academic and professional success of these future nurses.

For research advancement, longitudinal studies with broader and more diversified samples are suggested, deepening understanding of factors that modulate resilience and evaluating intervention effectiveness. Continuous investigation and strengthening of resilience among nursing students are crucial not only to ensure their successful training but also to enable them to provide high-quality care and deal with the complexities inherent to the profession, ensuring their own health and that of their future patients.

## Figures and Tables

**Table 1:** Overview of Included Studies.

Citation Number	Title	Publication Type & Country	Year of Publication	Databases from General Search Strategy
[12]	Estresse e resiliência em discentes de enfermagem de duas universidades públicas paulistas.	Peer-reviewed article, Brazil	2020	CINAHL, EMBASE, GOOGLE SCHOLAR, LILACS, MEDLINE, PUBMED, SCIELO, SCOPUS, WEB OF SCIENCE
[15]	Fatores sociodemográficos e acadêmicos relacionados à resiliência dos graduandos da área da saúde.	Peer-reviewed article, Brazil	2020	CINAHL, EMBASE, GOOGLE SCHOLAR, LILACS, MEDLINE, PUBMED, SCIELO, SCOPUS, WEB OF SCIENCE
[56]	Alterações de saúde, resiliência e qualidade de vida de discentes de graduação em enfermagem no primeiro ano letivo.	Doctoral Dissertation, Brazil	2017	CINAHL, EMBASE, GOOGLE SCHOLAR, LILACS, MEDLINE, PUBMED, SCIELO, SCOPUS, WEB OF SCIENCE
[57]	Resiliencia en los estudiantes del quinto ciclo de enfermería y psicología de la universidad autónoma de ICA, Lima, Peru.	Thesis, Peru	2019	CINAHL, EMBASE, GOOGLE SCHOLAR, LILACS, MEDLINE, PUBMED, SCIELO, SCOPUS, WEB OF SCIENCE
[58]	Resiliencia y sintomatología depresiva en estudiantes de enfermería de un Instituto Público, Lima, Peru.	Thesis, Peru	2023	CINAHL, EMBASE, GOOGLE SCHOLAR, LILACS, MEDLINE, PUBMED, SCIELO, SCOPUS, WEB OF SCIENCE
[59]	Estrés académico y resiliencia en estudiantes de internado en enfermería de una universidad privada, Lima, Peru.	Thesis, Peru	2023	CINAHL, EMBASE, GOOGLE SCHOLAR, LILACS, MEDLINE, PUBMED, SCIELO, SCOPUS, WEB OF SCIENCE

Note: Specific database return information for each individual study is available in Supplementary S4. The listed databases reflect the comprehensive search strategy employed to identify all studies. Source: Author.

## Data Availability

Data is contained within the article or Supplementary Materials.

## Supplementary Materials

The following supporting information can be downloaded at: https://www.mdpi.com/article/10.3390/ijerph22091425/s1, Supplementary S1: Search strategies for all databases searched; Supplementary S2: List of included studies and their characteristics; Supplementary S3: Data Extraction Tool; Supplementary S4: Tables summarizing the results.

## Abbreviations

The following abbreviations are used in this manuscript:

CES-D	Center for Epidemiologic Studies Depression Scale
CINAHL	Cumulative Index to Nursing and Allied Health Literature
DeCS	Health Sciences Descriptors
DOI	Digital Object Identifier
EMBASE	Excerpta Medica Database
JBI	Joanna Briggs Institute
LILACS	Latin American and Caribbean Literature in Health Sciences
MEDLINE	Medical Literature Analysis and Retrieval System Online
MeSH	Medical Subject Headings
OSF	Open Science Framework
PCC	Population, Concept, Context
PRISMA	Preferred Reporting Items for Systematic Reviews and Meta-Analyses
PRISMA-ScR	Preferred Reporting Items for Systematic Reviews and Meta-Analyses extension for Scoping Reviews
PSQI	Pittsburgh Sleep Quality Index
PubMed	Public/Publisher MEDLINE (da U.S. National Library of Medicine)
REDCap	Research Electronic Data Capture
SCIELO	Scientific Electronic Library Online
SCOPUS	Scopus database (Elsevier)
SEM	Structural Equation Modeling
SPSS	Statistical Package for the Social Sciences
WHOQOL-Bref	World Health Organization Quality of Life
WY-RS	Wagnild and Young Resilience Scale
