# Supplementary material for: Psychological Resilience in Latin America Nursing Students Using the Wagnild and Young Scale: A Scoping Review
Source: Int J Environ Res Public Health. 2025 Sep 12;22(9):1425. doi: 10.3390/ijerph22091425 (PMC12469408; doi:10.3390/ijerph22091425)
Supplement: Supplementary file 1 [file ijerph-22-01425-s001.zip › ijerph-3810696-supplementary.pdf]

Supplementary S1: Search strategies for all databases searched.

Table 1A. Subjects and synonyms used in the structuring of the search strategy.

**AFFAIRS**	**SUBJECT AND SYNONYMS IN ENGLISH (DeCS)**	**SUBJECT AND SYNONYMS IN ENGLISH (MeSH)**
SUBJECT MATTER 1	"Nursing Students" OR "Students" OR "Healthcare Personnel" OR "Nurses" OR "Nursing" OR "Nursing Students" OR "Student Nurses" OR "Student Nurses"	*"Students Nursing" OR "Students" OR "Health Personnel" OR "Nurses" OR "Nursing" OR "Nurses Students" OR "Nursing Students" OR "Student Nurses"*
SUBJECT MATTER 2	"Psychological Resilience"	*"Resilience, Psychological"*
SUBJECT MATTER 3	"Mental Health" OR "Mental Hygiene" OR "Mental Health Area"	*"Mental Health"*

Source: Authors (2025).

Table 1B. Databases used for searching.

**DATABASE**	**STRATEGY**	**NUMBER OF ITEMS FOUND**
**Search Strategy IN ENGLISH (EMBASE; MEDLINE; PUBMED; SCOPUS; WEB OF SCIENCE;**	*("Students Nursing" OR "Students" OR "Health Personnel" OR "Nurses" OR "Nursing" OR "Nurses Students" OR "Nursing Students" OR "Student Nurses" AND ("Resilience, Psychological") AND ("Mental Health")*	EMBASE (156)<br>MEDLINE (75)<br>PUBMED (82)<br>SCOPUS (39)<br>WEB OF SCIENCE (117)
**Search Strategy IN PORTUGUESE**<br>**GOOGLE SCHOLAR;**<br>**SCIELO**	("Nursing Students" OR "Students" OR "Healthcare Personnel" OR "Nurses" OR "Nursing" OR "Nursing Students" OR "Student Nurses" OR "Student Nurses" OR "Healthcare Professional") AND ("Psychological Resilience") AND ("Mental Health" OR "Mental Hygiene" OR "Mental Health Area")	GOOGLE SCHOLAR (97)<br>SCIELO (13)

Source: Authors (2025).

Table 2A. Subjects and synonyms used in the structuring of the search strategy.

**AFFAIRS**	**SUBJECT AND SYNONYMS IN PORTUGUESE (DeCS)**	**SUBJECT AND SYNONYMS IN ENGLISH (MeSH)**
SUBJECT MATTER 1	"Nursing Students" OR "Students" OR "Healthcare Personnel" OR "Nurses" OR "Nursing" OR "Nursing	*"Students Nursing" OR "Students" OR "Health Personnel" OR "Nurses" OR "Nursing" OR "Nurses Students"*

	Students" OR "Student Nurses" OR "Student Nurses"	*OR "Nursing Students" OR "Student Nurses"*
SUBJECT MATTER 2	"Psychological Resilience"	*"Resilience, Psychological"*
SUBJECT MATTER 3	"Mental Health" OR "Mental Hygiene" OR "Mental Health Area"	*"Mental Health"*

Source: Authors (2025).

Table 2B. Databases used for searching.

DATABASE	STRATEGY	NUMBER OF ITEMS FOUND
**Search Strategy IN ENGLISH (**Cumulative Index to Nursing and Allied Health Literature **(CINAHL);** National Library of Medicine **(PubMed); SCOPUS; WEB OF SCIENCE; MEDLINE and EMBASE**	*("Students Nursing" OR "Students" OR "Health Personnel" OR "Nurses" OR "Nursing" OR "Nurses Students" OR "Nursing Students" OR "Student Nurses" AND ("Resilience, Psychological") AND ("Mental Health")*	CINAHL (166)<br>PUBMED (128)<br>SCOPUS (183)<br>Web of Science (54)<br>Embase (50)<br>Medline (149)
**Search Strategy PORTUGUESE/ENGLISH** Scientific Electronic Library Online **(SCIELO); (**Cumulative Index to Nursing and Allied Health Literature **(CINAHL); Google Scholar**	("Nursing Students" OR "Students" OR "Healthcare Personnel" OR "Nurses" OR "Nursing" OR "Nursing Students" OR "Student Nurses" OR "Student Nurses" OR "Healthcare Professional") AND ("Psychological Resilience") AND ("Mental Health" OR "Mental Hygiene" OR "Mental Health Area")	Google Scholar (13)<br>CINAHL (20)<br>Scielo (50)

Source: Authors (2025).

Supplementary S2: List of included studies and their characteristics

1- Silva, R.M. ***Health changes, resiliency and quality of life of nursing degree students after the first academic-year*** **[Doctoral thesis]. University of São Paulo, School of Nursing; 2017. https://www.teses.usp.br/teses/disponiveis/7/7139/tde-08032018-134331/en.php**

• **Method:** Quantitative longitudinal study with a methodological calibration phase and structural equation modeling.

• **Sample:** 117 undergraduate nursing students in their first academic year at two public universities in São Paulo, Brazil.

• **Key findings:** The study revealed a significant increase in psycho-emotional stress and depressive symptoms, and a decline in sleep duration and subjective sleep quality over the academic year. The hypothesized model demonstrated that psycho-emotional stress decreased sleep quality and increased depressive symptoms, which directly and indirectly reduced quality of life. Resilience played a protective role, alleviating stress and depressive symptoms, and improving sleep quality.

• **Thematic analysis identified key themes:** (1) the academic nursing environment as a potential source of illness, (2) psycho-emotional stress as a central predictor of health decline, (3) resilience as a moderator mitigating stress effects, (4) structural equation modeling as a tool for understanding interrelated health phenomena, and (5) the need for health-promoting curricular strategies.

• The study calls for academic institutions to re-evaluate curricular demands and promote resilience and wellness among students. It highlights the importance of early interventions during nursing education to enhance students' mental health, resilience, and overall quality of life.

2- Mejia, J.A. ***Resilience in fifth-cycle nursing and psychology students at Universidad Autónoma de Ica, 2018*** **[Undergraduate thesis]. Universidad Autónoma de Ica, Faculty of Health Sciences; 2019. https://hdl.handle.net/20.500.14441/531**

• **Method:** Descriptive, cross-sectional, non-experimental design using the Wagnild and Young Resilience Scale.

• **Sample:** 47 undergraduate students (25 in Psychology and 22 in Nursing) enrolled in the fifth academic cycle at Universidad Autónoma de Ica, Peru.

• **Key findings:** The study found a medium level of resilience among nursing students and a low level among psychology students. In terms of resilience dimensions, nursing students scored highest in perseverance, while psychology students scored highest in the "feeling good alone" dimension. Most participants were female (78.72%) and single (87.23%). Age group 16–19 was the most represented (57.45%).

• **Thematic analysis identified key themes:** (1) the variation in resilience levels between

academic disciplines, (2) sociodemographic factors influencing resilience such as age and living arrangements, (3) dimension-specific strengths and weaknesses in resilience profiles, (4) gender distribution in health education fields, and (5) institutional responsibility in developing students' emotional resilience.

• The study recommends implementing university-wide workshops for students, staff, and parents to enhance resilience. It emphasizes the importance of further research on sociodemographic and contextual factors that influence resilience and calls for tailored interventions to strengthen psychological well-being in academic environments.

3- Moraes-Filho, I.M., Nascimento, F.A., Bastos, G.P., et al. Sociodemographic and academic factors related to health undergraduate students' resilience. *Revista de Divulgação Científica Sena Aires*, 9(2), 291–303 (2020). https://doi.org/10.36239/revisa.v9.n2.p291a303

• **Method:** Quantitative, cross-sectional, analytical study using a sociodemographic and academic questionnaire and the Wagnild & Young Resilience Scale.

• **Sample:** 138 health sciences undergraduate students (Nursing, Physiotherapy, and Pharmacy) from a private higher education institution in Goiás, Brazil.

• **Key findings:** 21.7% of students exhibited high resilience, and 71.7% moderate resilience. The most influential resilience factors were "Actions and Values Resolution" and "Self-confidence and Adaptability." Statistically significant associations were identified between resilience levels and variables such as leisure activity, satisfaction with the academic program, and living arrangements. Students who engaged in leisure activities, expressed satisfaction with their course, and lived with family members showed higher resilience.

• **Thematic analysis identified key themes:** (1) the multifactorial nature of resilience among health students, (2) social and environmental influences on psychological strength, (3) the role of satisfaction with one's academic path in resilience outcomes, (4) the importance of familial support, and (5) the need for structured institutional efforts to cultivate resilience.

• The study concludes that moderate to high resilience among health students is positively associated with leisure, academic satisfaction, and family support. These findings highlight the need for universities to implement well-being strategies and create educational environments conducive to developing student resilience.

4- **Souza, F.O., Silva, R.M., Costa, A.L.S., et al. Stress and resilience in nursing students from two public universities in São Paulo. *Revista de Enfermagem da UFSM*, 10, e2, 1–17 (2020). https://doi.org/10.5902/2179769234162**

• **Method:** Cross-sectional quantitative study.

• **Sample:** 117 first-year undergraduate nursing students from a federal and a state public university in São Paulo, Brazil.

• **Key findings:** The study revealed that 72.6% of the students experienced moderate stress, particularly regarding time management (23.9%) and theoretical activities (20.5%). Additionally,

11.1% reported very high stress related to the academic environment. Despite 51% of students exhibiting low resilience levels and 45% moderate resilience, no significant correlation was found between stress levels and resilience scores.

• **Thematic analysis identified key themes:** (1) nursing education as a potential source of stress and illness, (2) time management and theoretical workload as primary stressors, (3) resilience levels being generally low to moderate among students, (4) absence of significant association between stress and resilience, and (5) implications for curricular and institutional improvements.

• The study emphasizes the necessity for institutions to create supportive educational environments by adjusting curricular demands, fostering spaces for rest and social interaction, and implementing health promotion programs. Strengthening students' emotional and behavioral resilience is proposed as a strategy to prevent negative outcomes such as burnout and mental health deterioration.

5- Vela Salvador, V.C. ***Resilience and depressive symptomatology in nursing students at a public institute in Lima, 2023*** **[Undergraduate thesis]. Universidad Privada Norbert Wiener, Faculty of Health Sciences; 2023.** https://hdl.handle.net/20.500.13053/9902

• **Method:** Quantitative, descriptive, correlational, cross-sectional study using the Wagnild & Young Resilience Scale and the Patient Health Questionnaire-9 (PHQ-9).

• **Sample:** 202 nursing students from a public institute in the San Juan de Lurigancho district, Lima, Peru, selected by non-probabilistic sampling.

• **Key findings:** The study revealed a significant inverse correlation between resilience and depressive symptoms (Spearman's r, $p < 0.05$). Students with higher resilience scores—particularly in dimensions like perseverance and self-confidence—tended to report lower levels of depressive symptoms. Despite moderate levels of resilience in the overall sample, depressive symptoms were prevalent, especially among those with lower resilience and inadequate emotional coping mechanisms.

• **Thematic analysis identified key themes:** (1) the psychological vulnerability of nursing students in post-pandemic academic environments, (2) resilience as a protective factor against depressive symptoms, (3) the role of sociodemographic factors and personal attributes in emotional regulation, (4) the importance of early detection of mental health indicators, and (5) the need for institutional programs to promote emotional well-being and resilience.

• The study recommends the development of mental health programs tailored to nursing students, incorporating strategies to strengthen emotional resilience, prevent depressive disorders, and promote academic and personal success. These initiatives are considered vital to protecting students' professional development and psychologic

6- Yesenia, J. B. G. ***Academic stress and resilience in nursing internship students at a private university, Lima 2023*** **[Undergraduate thesis]. Universidad Norbert Wiener, Faculty of Health Sciences; 2023.** https://hdl.handle.net/20.500.13053/10046

• **Method:** Quantitative, non-experimental, correlational, and descriptive study using a hypothetical-deductive method.

• **Sample:** 95 nursing internship students from a private university in Lima, Peru, selected by intentional non-probabilistic sampling during May 2023.

• **Key findings:** The study found a statistically significant relationship between academic stress and resilience (p=0.000). Although 70.5% of the participants reported low academic stress and 63.2% demonstrated high resilience, the findings suggest that resilience acts as a buffer to stress in academic settings. Higher resilience scores correlated with lower stress symptoms and improved coping strategies.

• **Thematic analysis identified key themes:** (1) the prevalence of academic stress during internships, (2) the protective role of resilience against physical and emotional stress symptoms, (3) the correlation between coping strategies and resilience, (4) gender and work status as potential influencing factors, and (5) the importance of institutional support in promoting student resilience.

• The study recommends integrating resilience-building strategies into nursing curricula, emphasizing stress management education and the creation of supportive environments to mitigate academic pressures and foster student well-being.

Supplementary S3: Data Extraction Tool

SECTION	DATA TO BE EXTRACTED
General Information	- Authors<br>- Year of publication<br>- Country of origin of the study<br>- Language of the article<br>- Type of publication (article, thesis, dissertation, etc.)<br>- Study objectives
Methodological Characteristics	- Study design (cross-sectional, longitudinal, etc.)<br>- Approach (quantitative, qualitative, mixed methods)<br>- Sampling procedures<br>- Sample size<br>- Inclusion and exclusion criteria<br>- Variables analyzed<br>- Data collection instruments used<br>- Data analysis procedures
Sample Characteristics	- Age<br>- Gender<br>- Year/period of the academic program<br>- Educational institution<br>- Other relevant characteristics
Results - Wagnild & Young Resilience Scale	- Overall mean score on the scale<br>- Mean scores for each factor/domain of the scale<br>- Classification of resilience levels (low, moderate, high)<br>- Percentage of students at each resilience level<br>- Comparisons of resilience levels among subgroups (gender, year of study, etc.)<br>- Correlations between resilience and other variables
Other Relevant Results	- Identified predictors of resilience<br>- Impacts of resilience on students' well-being and mental health<br>- Strategies discussed for promoting resilience

SECTION	DATA TO BE EXTRACTED
	- Identified gaps and recommendations for future research
Quality Assessment and Limitations	- Description of strengths and limitations as reported by the authors<br>- Assessment of potential risk of bias (selection bias, information bias, etc.)<br>- Analysis of the adequacy of statistical methods<br>- Considerations regarding the internal and external validity of the study
Conclusions and Implications	- Summary of the main conclusions drawn by the authors<br>- Discussion on the implications of the findings for nursing practice, education, and research<br>- Recommendations for interventions or strategies to promote resilience based on the evidence

This instrument aims to comprehensively map the key aspects to be extracted from the studies, considering the specificities of the topic (resilience in nursing students) and the measurement tool used (Wagnild & Young Resilience Scale).

Systematic extraction of these data will enable a detailed characterization of the included studies, allowing for in-depth analyses of the levels of resilience found, associated factors, implications, and gaps in the existing literature.

Supplementary S4: Tables summarizing the results

Table 1. General Study Information

Study ID	Authors	Publication Year	Country of Origin	Language	Publication Type (article, thesis, dissertation, etc.)	Study Objectives
01/2017	Silva, R.M.	2017	Brazil	Portuguese	Doctoral thesis	• To ascertain whether significant alterations occur in the health status, resilience levels, and quality of life of nursing undergraduate students following their first academic year.<br>• To analyze the intricate simultaneous causal relationships among psychoemotional stress, depressive symptoms, sleep quality, and resilience in explaining the quality of life among nursing students during their inaugural academic year.

02/2019	Mejía, J.A. de	2019	Peru	Spanish	Undergraduate thesis (Bachelor of Psychology)	To determine the level of resilience among fifth-semester students from the nursing and psychology programs at a Peruvian university during the second academic semester of 2018.
03/2020	Moraes-Filho, M. de, Nascimento, F.A. do N., Bastos, G.P., Júnior F.E. de S.B., Silva, R.M. da, Santos, A.L.M., Abreu, C.R. de C., Valóta, I.A. das C.	2020	Brazil	Portuguese	Peer-reviewed scientific article	To analyze the association between sociodemographic and academic factors and the resilience levels of undergraduate students in health-related academic programs.
04/2020	Souza, F.O. de, Silva, R.M da, Costa, A.L.S., Mussi, F.C., Santos, C.C.T., Santos, O.P. dos	2020	Brazil	Portuguese	Peer-reviewed scientific article	To investigate the relationship between stress and resilience among undergraduate nursing students

						enrolled at two public universities in the state of São Paulo.
05/2023	Salvador, V.C.V.	2023	Peru	Spanish	Undergraduate thesis in Psychology	General Objective: To determine the relationship between resilience and depressive symptomatology in nursing students from a public institute.<br><br>Specific Objectives: To assess the relationship between perseverance and depressive symptoms.<br><br>To examine the relationship between self-confidence and depressive symptoms.

						To evaluate the association between personal satisfaction and depressive symptoms.<br><br>To analyze the relationship between equanimity and depressive symptoms.<br><br>To investigate the relationship between comfort with solitude and depressive symptoms.
06/2023	Yesenia, J.B.G	2023	Peru	Spanish	Undergraduate thesis	• To determine the relationship between academic stress and resilience among nursing internship students at a private university.<br>• To analyze the relationship between academic

						stress (in its stressor, symptom, and coping strategy dimensions) and resilience among nursing interns.

Table 2. Methodological Characteristics

Study ID	Study Design (cross-sectional, longitudinal, etc.)	Approach (quantitative, qualitative, mixed methods)	Sampling procedures	Sample size	Inclusion and exclusion criteria	Variables analyzed	Data collection instruments used	Data analysis procedures
01/2017	The research employed a quantitative approach, structured into two distinct phases: a methodological phase focused on instrument calibration and a prospective longitudinal phase. The methodological segment aimed to investigate data collection and organization methods to facilitate tool evaluation and refinement. The longitudinal component involved tracking a cohort of participants over	The study's methodology is exclusively quantitative.	A non-probability, convenience sampling technique was utilized, enrolling students who voluntarily consented to participate.	• Phase 1 (Calibration): The calibration phase involved 110 nursing students. • Phase 2 (Longitudinal): For the longitudinal phase, 117 students were initially enrolled in March, with a subsequent participation of 100 students in December 2016. Notably, 84 students provided data for both collection points, and an additional 16	• Phase 1 (Calibration): Inclusion: Participants were required to be regularly enrolled second, third, or fourth-year nursing students (corresponding to the 4th, 6th, and 8th academic semesters, respectively) at Institution A during the second semester of 2015, and aged 18 years or older. Exclusion: Students who,	• For assessing changes in health, resilience, and quality of life: The academic environment of the nursing undergraduate program served as the primary predictor variable. Psychoemotional stress, depressive symptoms, sleep quality, resilience, and quality of life were designated as outcome variables. • For examining the dynamics of health phenomena: Psychoemotional stress was established as the	• A sociodemographic and academic characterization form. • The Instrument for Stress Evaluation in Nursing Students (AEEE). • The Center for Epidemiologic Studies Depression Scale (CES-D). • The Pittsburgh Sleep Quality Index (IQSP). • The Wagnild & Young Resilience Scale. • The World Health Organization Quality of Life Instrument –	• General Procedures: Qualitative variables were quantified and presented as absolute numbers (n) and percentages (n%). Quantitative variables were summarized using descriptive statistics, including minimum and maximum values, mean, and standard deviation. Instrument reliability was assessed using

	time to measure variables capable of predicting future outcomes.			new students were incorporated during the second collection.	at the time of data collection, were unable to enroll in all subsequent semester courses due to academic failures in the preceding semester were excluded. • Phase 2 (Longitudinal): Inclusion: Eligible participants were first-year nursing students (enrolled in the 1st and 2nd semesters of 2016) at both Institution A and Institution B, aged 18 years or older. Exclusion: Analogous to Phase 1, students unable to	predictor variable, with quality of life as the ultimate outcome variable. Resilience, depressive symptoms, and sleep quality were identified and treated as mediating variables within the model.	Abbreviated Version (WHOQOL-Bref).	Cronbach's Alpha coefficient. • Phase 1 (Calibration): Confirmatory Factor Analysis (CFA) was conducted using the Diagonally Weighted Least Squares (DWLS) estimation method. Model fit indicators included Chi-square (χ2), Normalized Chi-square (χ2/df), Goodness-of-Fit Index (GFI), Comparative Fit Index (CFI), Tucker-Lewis Index (TLI), Standardized Root Mean Square Residual (SRMR), and

					enroll in all subsequent semester courses due to prior academic failures were excluded.			Root Mean Square Error of Approximation (RMSEA). Factor loadings and Pearson correlations were also analyzed. • Phase 2 (Longitudinal): Changes in variables over time were assessed using ANOVA for mixed models. To address the final objective, Multivariate Analysis, specifically Structural Equation Modeling (SEM), was employed. This technique utilized the same estimation method and fit indices as the

								CFA. Mediation analysis involved confirming significant relationships among predictor, mediator, and outcome variables, followed by a comparison of direct and mediated effects.
02/2019	Non-experimental, descriptive, and cross-sectional design	Quantitative	Convenience sampling involving all students enrolled in the fifth semester of nursing and psychology during the specified academic period	47 students (25 from psychology and 22 from nursing)	No information was found regarding this item in the reviewed source.	Main variable: Resilience (including dimensions such as self-confidence, equanimity, perseverance, personal satisfaction, and comfort with solitude)<br>Sociodemographic variables: age, gender, marital	A self-developed sociodemographic questionnaire<br>The Wagnild & Young Resilience Scale (1993), composed of 25 items distributed across five dimensions; scoring range: 25–175; classifications: low (25–75), moderate (76–	Data were processed using IBM SPSS version 25. Results were presented using descriptive statistics (frequencies, percentages, tables, and figures).

						status, academic field	125), high (126–175)	
03/2020	Cross-sectional and analytical study	Quantitative	Non-probabilistic sampling by convenience. Participants were recruited in classrooms after prior arrangement with course instructors. Data collection occurred through self-administered instruments.	138 undergraduate students from Nursing, Physiotherapy, and Pharmacy programs	Inclusion: students aged 18 years or older enrolled in health-related undergraduate programs<br><br>Exclusion: students participating in academic exchange programs	Sociodemographic and academic characteristics, resilience levels	Sociodemographic and academic questionnaire<br>Wagnild & Young Resilience Scale (adapted to the Brazilian context)	Data were processed using SPSS v16.0. Descriptive statistics, chi-square tests, and significance levels of $p < 0.05$ were applied. Categorical variables were reported as absolute and relative frequencies; ordinal/interval variables were described using mean, minimum and maximum values, and standard deviation.

04/2020	Cross-sectional study	Quantitative	The study included all first-year nursing students from a public state university and a federal university, who voluntarily agreed to participate by signing an informed consent form.	117 undergraduate students	Inclusion: Students aged 18 years or older, regularly enrolled in the first year of their nursing program.<br><br>Exclusion: Students who were unable to enroll in all courses for the subsequent semester due to academic failures.	Sociodemographic and academic characteristics, stress levels, resilience levels	Academic and sociodemographic questionnaire<br>Nursing Students Stress Assessment Instrument (AEEE)<br>Wagnild & Young Resilience Scale	Data were analyzed using SPSS version 10.0. Descriptive statistics, Cronbach's alpha for reliability analysis, and Pearson's correlation test were used. A significance level of $p < 0.05$ was adopted.
05/2023	Cross-sectional correlational study	Quantitative	Non-probabilistic sampling	202 participants	Inclusion: Nursing students enrolled at a public technical institute in Lima	Independent variable: Resilience<br>Dependent variable: Depressive symptomatology	Wagnild & Young Resilience Scale (adapted by Novella, 2002)<br>Patient Health Questionnaire-9 (PHQ-9)	Descriptive statistics, normality tests, and Spearman's correlation coefficient

					Exclusion: No information found			
06/2023	Cross-sectional, observational, non-experimental, correlational	Approach: Quantitative	Sampling procedures: Census sampling; non-probabilistic and intentional selection	Sample size: 95 students	Inclusion criteria: Nursing internship students over 18 years of age, of any gender, regularly attending classes, and who signed the informed consent form.<br><br>Exclusion criteria: Students diagnosed with mental health disorders, those who failed to submit the questionnaires within the deadline, or those on	Academic stress: stressors, symptoms, and coping strategies<br><br>Resilience: five dimensions based on the Wagnild & Young scale	Academic Stress Inventory (SISCO)<br><br>Wagnild & Young Resilience Scale	Descriptive and inferential statistical análisis<br><br>Pearson correlation<br><br>Hypothesis testing

					medical or personal leave.			

Table 3. Sample Characteristics

Study ID	Age	Gender	Year/period of the academic program	Educational institution	Other relevant characteristics
01/2017	• Calibration (Institution A): The mean age of participants was 21.87 years (SD ±3.26).<br>• Longitudinal (Institutions A and B): The mean age was 20.73 years (SD ±4.4) in March 2016, slightly increasing to 20.90 years (SD ±5.1) by December 2016.	• Calibration (Institution A): Females constituted 92.7% of the sample.<br>• Longitudinal (Institutions A and B): Females comprised 84.6% in March 2016 and 88.0% in December 2016.	• Phase 1 (Calibration): The study recruited students from the second, third, and fourth years of their academic program (corresponding to the 4th, 6th, and 8th semesters).<br>• Phase 2 (Longitudinal): This phase focused on first-year students (enrolled in the 1st and 2nd semesters of 2016).	• Phase 1 (Calibration): The study was conducted at a single state university in São Paulo, designated as Institution A.<br>• Phase 2 (Longitudinal): This phase expanded to include participants from both a state university (Institution A) and a federal university (Institution B), both situated within the state of São Paulo.	• Marital Status: A predominant proportion of the sample was single and without a partner (83.8% in March; 78% in December).<br>• Children: The vast majority of participants reported having no children (95.7% in March; 98% in December).<br>• Residency: Most students resided in São Paulo (85.5% in March; 83% in December), primarily with family (80.3% in March; 81% in December), and in their own apartment or house (65.8% in March; 65% in December).<br>• High School Background: Over half of the students attended private high schools (51.3% in March; 52% in December).<br>• Sports Participation: A majority did not engage in sports activities (65.8% in March; 76% in December).<br>• Leisure Activities: Most reported participating in leisure activities (60.7% in March; 61% in December).<br>• Income Source: Financial support primarily stemmed from family resources (69.2% in March; 66% in December).<br>• Sleep-Inhibiting Medication Use: A notable percentage used medication to inhibit sleep (57.3% in March; 52% in December).<br>• Sleep-Inducing Medication Use: The majority did not use medication to aid sleep (88.9% in March; 85% in December).

					• Smoking Status: Most participants reported never smoking (91.5% in March; 88% in December).<br>• Alcohol Consumption: A significant portion consumed alcoholic beverages (63.2% in March; 65% in December), typically on a monthly or weekly basis.<br>• Commute Time to Institution: The average daily commute to the educational institution was approximately one hour.<br>• Employment Status: The majority of students were not employed (93.2% in March; 89% in December).<br>• Course Satisfaction: A high level of satisfaction with their nursing program was observed (88.0% in both March and December).<br>• Intent to Drop Out: The percentage of students considering dropping out decreased from 64.1% in March to 53.0% in December.<br>• Daily Study Hours: Daily study hours notably increased from 2.7 hours in March to 6.0 hours in December.<br>• Extracurricular Activities: Participation in extracurricular activities slightly decreased from 56.4% in March to 51.0% in December.
02/2019	Ages ranged from 16 to 31; the majority (57.45%) were between 16 and 19 years old	78.72% female, 21.28% male	Fifth semester	Universidad Autónoma de Ica	• 87.23% were single<br>• 53.19% were enrolled in psychology, and 46.81% in nursing
03/2020	No information was found regarding this	75.4% female	31.9% were in the fifth semester	Private higher education institution	• 73.2% single

	item in the reviewed source.			located in the metropolitan area of Brasília, Brazil	• 68.1% had no children<br>• 89.1% lived with family<br>• 90.6% engaged in leisure activities<br>• 52.9% had a fixed income from work<br>• 99.3% were satisfied with their academic program
04/2020	Mean = 20.73 years (SD = 4.4)	84.6% female	First year of the undergraduate nursing course	One public state university and one public federal university located in São Paulo, Brazil	Most participants were single (83.8%), had no children (95.7%), did not engage in sports (65.8%), but reported participating in leisure activities (60.7%). The average daily study time was 2.7 hours, with a mean of 11 enrolled courses. Most did not work (93.2%) and engaged in extracurricular activities (56.4%). The mean semester workload was 442.9 hours at Institution A and 848.6 hours at Institution B.
05/2023	Majority between 18 and 25 years old	82% female, 18% male	Data on academic module/year were collected but not specified in detail	Public technical institute located in San Juan de Lurigancho, Lima	No information found
06/2023	Mean age of participants: 30.6 years	89.4% female	Final-year undergraduate students enrolled in the nursing internship program	Norbert Wiener University, Lima, Peru	• 51.6% were single<br>• 88.4% were employed at the time of the study

Table 4. Results - Wagnild & Young Resilience Scale

Study ID	Overall mean score on the scale	Mean scores for each factor/domain of the scale	Classification of resilience levels (low, moderate, high)	Percentage of students at each resilience level	Percentage of students at each resilience level	Comparisons of resilience levels among subgroups (gender, year of study, etc.	Correlations between resilience and other variables
01/2017	The overall mean resilience score was 120.36 in March and 119.86 in December, indicating no statistically significant change over the period.	• March 2016: Action and Values: 5.06<br>✓ Independence and Determination: 3.90<br>✓ Self-Confidence and Adaptability: 5.22<br>• December 2016:<br>✓ Action and Values: 4.97<br>✓ Independence and Determination: 4.00<br>✓ Self-Confidence and Adaptability: 5.23<br>✓ No statistically significant alterations were observed in the mean scores across any of the resilience factors.	Based on the average overall scores (120.36 in March and 119.86 in December), participants generally fall at the threshold between "reduced resilience" (below 121) and "moderate resilience" (between 121 and 145), leaning towards "reduced resilience" by the end of the academic year.	NO INFORMATION ABOUT THIS ELEMENT WAS FOUND IN THIS SOURCE	NO INFORMATION ABOUT THIS ELEMENT WAS FOUND IN THIS SOURCE	NO INFORMATION ABOUT THIS ELEMENT WAS FOUND IN THIS SOURCE	Resilience demonstrated a mitigating effect on psychoemotional stress (β=-0.51) and depressive symptoms (β=-2.75), in addition to enhancing sleep quality (β=-0.17), thereby acting as a protective factor. However, resilience did not exhibit a direct effect on the students' overall quality of life.

02/2019	No information was found regarding this item in the reviewed source.	No numerical mean scores reported; only distribution by level (low, moderate, high)	• Nursing students: predominantly moderate level of resilience<br>• Psychology students: predominantly low level of resilience	NO INFORMATION ABOUT THIS ELEMENT WAS FOUND IN THIS SOURCE	No global percentages were reported; only breakdowns by dimension and academic field	• Nursing students scored highest in perseverance<br>• Psychology students scored highest in the dimension "feeling good alone"	No information was found regarding this item in the reviewed source.
03/2020	No information was found regarding this item in the reviewed source.	• Actions and Values: Mean = 5.88 (SD = 0.44)<br>• Independence and Determination: Mean = 4.97 (SD = 0.20)<br>• Self-confidence and Adaptability: Mean = 5.87 (SD = 0.77)	• Low resilience: 6.1%<br>• Moderate resilience: 71.7%<br>• High resilience: 21.7%	NO INFORMATION ABOUT THIS ELEMENT WAS FOUND IN THIS SOURCE	As reported above	Statistically significant associations were found between resilience levels and:<br>• Living arrangements (p = 0.003)<br>• Engagement in leisure activities (p = 0.010)<br>• Satisfaction with the	Resilience was positively correlated with living with family, engaging in leisure activities, and being satisfied with the academic program.

						academic program (p = 0.001)	
04/2020	No information was found regarding this item in the reviewed source.	The domain "Actions and Values" had the highest contribution to resilience, with a mean score of 5.06 (SD = 0.84).	Low resilience: 51%<br>Moderate resilience: 45%	NO INFORMATION ABOUT THIS ELEMENT WAS FOUND IN THIS SOURCE	Low: 51%<br>Moderate: 45%<br>(High resilience level not explicitly reported)	No information was found regarding this item in the reviewed source.	No significant correlations were found between overall or domain-specific stress scores and resilience scores.
05/2023	No information found	No information found	Reported qualitatively (low, moderate, high), but no specific percentages provided	NO INFORMATION ABOUT THIS ELEMENT WAS FOUND IN THIS SOURCE	No information found	No information found	Statistically significant negative correlation between resilience and depressive symptomatology (Spearman's rho)
06/2023	Information not available in the reviewed source	Information not available in the reviewed source	o High resilience: 63.2% of participants	o 63.2% reported high resilience	Percentages for other levels were not specified	• Comparisons of resilience levels among subgroups: Information not available in the reviewed source	• Correlations between resilience and other variables:<br>o Statisticall

							y significant correlations were found between overall academic stress and resilience (p = 0.000)<br>o Significant correlations were also observed between resilience and the following dimensions of academic stress:<br>Stressors (p = 0.08)<br>Symptoms (p = 0.000)<br>Coping strategies (p = 0.000)

Table 5. Other Relevant Results

Study ID	Identified predictors of resilience	Impacts of resilience on students' well-being and mental health	Strategies discussed for promoting resilience	Identified gaps and recommendations for future research
01/2017	NO INFORMATION ABOUT THIS ELEMENT WAS FOUND IN THIS SOURCE	Resilience functions as a crucial protective factor, effectively minimizing levels of psychoemotional stress, reducing the intensity of depressive symptoms, and improving sleep quality among nursing students. By enabling students to perceive stressors as challenges and adopt adaptive behaviors, resilience contributes to enhanced overall academic performance and general health status.	The study advocates for the development and implementation of training programs specifically designed to bolster emotional, mental, and behavioral skills, thereby fostering resilience in nursing students. It suggests that such programs, already successfully applied in high school settings, warrant further exploration and adaptation to the unique contextual factors influencing resilience within the university environment.	• Identified Gaps in Literature:<br>✓ A recognized scarcity of longitudinal health analyses following entry into nursing undergraduate programs.<br>✓ Limited research on resilience among university students in Brazil, particularly within nursing cohorts.<br>✓ An absence of studies examining simultaneous causal relationships among psychoemotional stress, depressive symptoms, sleep quality, quality of life, and resilience within nursing education.<br>✓ Insufficient research evaluating sleep quality in young adults undergoing academic training in Brazil.<br>✓ Underutilization of Structural Equation Modeling (SEM) as a research methodology in nursing studies.<br>• Recommendations for Future Research:

				✓ Conduct further applications of factor analysis in populations with larger sample sizes to improve the psychometric properties of the Wagnild & Young Resilience Scale.<br>✓ Encourage more frequent use of construct validity analysis for already validated instruments, especially when applied to distinct populations or when original validation dates are older than two years.<br>✓ Promote in-depth studies on resilience within health training contexts to better understand related factors and inform the creation of targeted interventions.<br>✓ Advocate for extended follow-up periods (beyond one year) for nursing students concerning sleep quality, quality of life, and resilience, recognizing that these phenomena may require more

				prolonged observation to manifest significant changes.
02/2019	No information was found regarding this item in the reviewed source.	The study emphasizes that resilience plays a protective role in academic and emotional well-being and may influence students' decision to persist or abandon their studies.	The study recommends organizing workshops and resilience-focused interventions for students, university staff, and families to strengthen adaptive capacities.	Future research should include larger, more heterogeneous samples and explore a broader range of individual, family, and community-level variables related to resilience.
03/2020	Leisure engagement, family cohabitation, and course satisfaction	Resilience contributed to effective coping with stressors in academic life and was suggested to enhance quality of life and emotional regulation	Encouragement of leisure activities, supportive family environments, and academic program satisfaction are discussed as resilience-strengthening strategies	No information was found regarding this item in the reviewed source.
04/2020	No information was found regarding this item in the reviewed source.	Although not statistically confirmed, the discussion emphasizes that resilience may act as a protective factor against academic stress, anxiety, depression, and burnout.	The article suggests institutional strategies such as curriculum revision, creation of wellness and leisure spaces, implementation of multiprofessional support teams, and development of emotional, behavioral, and mental resilience training programs for health students.	The study highlights a lack of research on resilience among nursing students in Brazil and recommends further studies involving private institutions, smaller cities, and students from other health-related programs.

05/2023	No information found	Students with higher levels of resilience tend to report fewer depressive symptoms	No information found	Recommends developing institutional interventions to support mental health among technical students
06/2023	Information not available in the reviewed source	Information not available in the reviewed source	Information not available in the reviewed source	o The study highlights a lack of national literature on the topic, emphasizing the need for further research involving larger and more diverse student populations across institutions

Table 6. Quality Assessment and Limitations

Study ID	Description of strengths and limitations as reported by the authors	Assessment of potential risk of bias (selection bias, information bias, etc.)	Analysis of the adequacy of statistical methods	Considerations regarding the internal and external validity of the study
01/2017	• Limitations:<br>✓ The study acknowledged a lack of detailed descriptions concerning the procedures for construct validity and reliability analyses of the utilized instruments.<br>✓ The scarcity of longitudinal studies involving nursing students posed a challenge for comparative analysis of findings.<br>✓ The necessity to respecify the Wagnild & Young Resilience Scale from a multifactorial to a unifactorial model, owing to suboptimal fit indices, indicates a future need for internal structure revision with larger sample sizes.<br>✓ A one-year follow-up period may have been insufficient to identify	NO INFORMATION ABOUT THIS ELEMENT WAS FOUND IN THIS SOURCE	The study judiciously applied Confirmatory Factor Analysis (CFA) and Structural Equation Modeling (SEM), methodologies deemed appropriate for investigating latent variables and complex interrelationships. The selection of the DWLS estimator and the chosen fit indices aligned with standard practices for such analyses. The favorable fit indices obtained for the final model substantiate the congruence between the hypothesized theoretical model and the empirical data.	NO INFORMATION ABOUT THIS ELEMENT WAS FOUND IN THE SOURCE READ

	substantial changes in sleep quality, resilience, and quality of life.			
02/2019	No information was found regarding this item in the reviewed source.	No information was found regarding this item in the reviewed source.	The statistical approach (descriptive analysis using SPSS) was appropriate for the objectives of the study.	No information was found regarding this item in the reviewed source.
03/2020	No information was found regarding this item in the reviewed source.	No information was found regarding this item in the reviewed source.	The use of chi-square tests and descriptive statistics was appropriate for the study objectives	No information was found regarding this item in the reviewed source.
04/2020	Reported limitations include the limited availability of studies on resilience among nursing students and the restricted context of participants from large public institutions in metropolitan areas.	No information was found regarding this item in the reviewed source.	Statistical methods used were appropriate and included validated instruments, reliability testing (Cronbach's alpha), and Pearson correlation.	No information was found regarding this item in the reviewed source.
05/2023	No information found	No information found	The study employed appropriate statistical tools (e.g., Spearman's	No information found

			test, descriptive statistics)	
06/2023	o The study acknowledges limited comparative literature and emphasizes that the cross-sectional design does not permit causal inferences, only associative ones	Information not available in the reviewed source	o The statistical methods applied, including Pearson correlation and hypothesis testing, were appropriate for the study's objectives and design	Information not available in the reviewed source

Table 7. Conclusions and Implications

Study ID	Authors' Main Conclusions	Implications (Practice, Education, Research)	Recommendations for Interventions/Strategies
01/2017	• The instruments employed demonstrated satisfactory construct validity and reliability for assessing the investigated phenomena in nursing students.<br>• Following the first academic year, a significant elevation in psychoemotional stress was observed, alongside a reduction in subjective sleep quality and an increase in the intensity of depressive symptoms (both overall and within the Somatic/Initiative domain). However, no significant changes were noted in overall quality of life, general sleep quality, or resilience.<br>• Psychoemotional stress was found to diminish sleep quality and intensify depressive symptoms, with the latter directly contributing to a reduction in quality of life. Depressive symptoms also served as a mediator, enabling an indirect effect of psychoemotional stress on quality of life.	• Implications for Nursing Education: Educational institutions are urged to critically re-evaluate their curricular elements to alleviate academic overload and address challenges related to time management and professional communication. It is recommended that they foster resilience and establish environments conducive to student health promotion, including regular psychological assessments for early identification of emerging disorders.<br>• Implications for Nursing Practice: The findings underscore the importance of cultivating healthier and better-prepared professionals capable of navigating the demands of the workforce. Such preparedness extends to empowering these future nurses to share their knowledge and coping strategies with other healthcare professionals and patients facing difficult circumstances.<br>• Implications for Research: A continued need for further longitudinal studies, new applications of factor analysis for resilience instruments, and in-depth investigations into resilience	• Implement strategies aimed at promoting resilience in nursing students, acknowledging its proven capacity to reduce psychoemotional stress, decrease the intensity of depressive symptoms, and enhance sleep quality.<br>• Assess the feasibility of establishing multidisciplinary support spaces within nursing schools dedicated to student health, focusing on disease prevention and health promotion.<br>• Consider the creation of dedicated rest areas within institutions, equipped with resources such as computers, sofas, beds, and gaming facilities, to allow students to rest and relax during their extensive hours on campus.

	• Resilience was shown to mitigate psychoemotional stress levels, enhance sleep quality, and lessen the intensity of depressive symptoms.<br>• The nursing training environment inherently possesses the potential to induce illness and distress, which can subsequently impact students' quality of life.<br>• Psychoemotional stress was identified as a strong predictor of health alterations, while resilience emerged as a protective factor against both stress and its associated negative outcomes.	within healthcare contexts is highlighted.	
02/2019	• Nursing students demonstrated a moderate level of resilience<br>• Psychology students demonstrated a low level of resilience<br>• Nursing students scored highest in perseverance, while psychology students scored highest in comfort with solitude	Understanding resilience levels among students is essential for fostering mental health and academic engagement. Institutions should implement strategies to monitor and enhance student resilience.	It is recommended to offer targeted resilience workshops and interventions involving students, staff, and families as part of an institutional approach to mental health and academic success.

03/2020	Undergraduate students in health-related fields demonstrated moderate to high levels of resilience. Key factors associated with higher resilience levels were engagement in leisure activities, satisfaction with the academic program, and living with family members.	Fostering resilience in health students can improve quality of life and academic performance. Institutions should promote environments that support emotional well-being and resilience development.	Academic institutions are encouraged to implement programs that promote student well-being, leisure, and satisfaction with the educational process.
04/2020	Moderate stress levels predominated among students. Time management and theoretical coursework were the most stressful factors. Most students presented low or moderate resilience, with no significant correlation observed between stress and resilience.	The study underscores the need for academic institutions to foster healthier educational environments, address curricular demands, and provide psychological support mechanisms to protect students' mental well-being.	Recommendations include implementing institutional programs focused on emotional education, promoting resilience development, and early identification of stress-related psychological risks among nursing students.
05/2023	A significant inverse relationship was observed between resilience and depressive symptoms. Higher resilience levels were associated with lower levels of depressive symptomatology among students.	The findings highlight the importance of incorporating resilience-building strategies into technical nursing education to mitigate mental health risks.	Institutional mental health support and early intervention are advised to enhance resilience and reduce depressive outcomes among students.

06/2023	o A significant relationship was found between academic stress and resilience among nursing internship students	o Resilience appears to act as a protective factor against academic stress<br>o Nursing programs should integrate emotional support systems and coping strategy development into their curricula	o The study recommends the implementation of structured emotional and psychological support strategies to strengthen students' resilience during training

Preferred Reporting Items for Systematic reviews and Meta-Analyses extension for Scoping Reviews (PRISMA-ScR) Checklist

SECTION	ITEM	PRISMA-ScR CHECKLIST ITEM	REPORTED ON PAGE #
**TITLE**			
Title	1	Identify the report as a scoping review.	1
**ABSTRACT**			
Structured summary	2	Provide a structured summary that includes (as applicable): background, objectives, eligibility criteria, sources of evidence, charting methods, results, and conclusions that relate to the review questions and objectives.	1-2
**INTRODUCTION**			
Rationale	3	Describe the rationale for the review in the context of what is already known. Explain why the review questions/objectives lend themselves to a scoping review approach.	2-4
Objectives	4	Provide an explicit statement of the questions and objectives being addressed with reference to their key elements (e.g., population or participants, concepts, and context) or other relevant key elements used to conceptualize the review questions and/or objectives.	4 (Section 2.2)
**METHODS**			
Protocol and registration	5	Indicate whether a review protocol exists; state if and where it can be accessed (e.g., a Web address); and if available, provide registration information, including the registration number.	4 (Section 2.1)
Eligibility criteria	6	Specify characteristics of the sources of evidence used as eligibility criteria (e.g., years considered, language, and publication status), and provide a rationale.	6–7 (Sections 2.4 and 2.5)
Information sources*	7	Describe all information sources in the search (e.g., databases with dates of coverage and contact with authors to identify additional sources), as well as the date the most recent search was executed.	6 (Section 2.3)
Search	8	Present the full electronic search strategy for at least 1 database, including any limits used, such that it could be repeated.	6–7 + Appendix 1
Selection of sources of evidence†	9	State the process for selecting sources of evidence (i.e., screening and eligibility) included in the scoping review.	7 + PRISMA-ScR Flowchart Figure 1
Data charting process‡	10	Describe the methods of charting data from the included sources of evidence (e.g., calibrated forms or forms that have been tested by the team before their use, and whether data charting was done independently or in duplicate) and any processes for obtaining and confirming data from investigators.	7-8 (Section 2.6)
Data items	11	List and define all variables for which data were sought and any assumptions and simplifications made.	7-8 (Section 2.6)
Critical appraisal of individual	12	If done, provide a rationale for conducting a critical appraisal of included sources of evidence; describe	13–17 (Discussion,

SECTION	ITEM	PRISMA-ScR CHECKLIST ITEM	REPORTED ON PAGE #
sources of evidence§		the methods used and how this information was used in any data synthesis (if appropriate).	study limitations)
Synthesis of results	13	Describe the methods of handling and summarizing the data that were charted.	8 (Section 2.7)
**RESULTS**			
Selection of sources of evidence	14	Give numbers of sources of evidence screened, assessed for eligibility, and included in the review, with reasons for exclusions at each stage, ideally using a flow diagram.	7 (PRISMA-ScR Flowchart Figure 1)
Characteristics of sources of evidence	15	For each source of evidence, present characteristics for which data were charted and provide the citations.	8–13
Critical appraisal within sources of evidence	16	If done, present data on critical appraisal of included sources of evidence (see item 12).	13–17 (Discussion, limitations)
Results of individual sources of evidence	17	For each included source of evidence, present the relevant data that were charted that relate to the review questions and objectives.	13–17 (Results + Table 1, Appendix 4)
Synthesis of results	18	Summarize and/or present the charting results as they relate to the review questions and objectives.	8–17 (Results + Discussion)
**DISCUSSION**			
Summary of evidence	19	Summarize the main results (including an overview of concepts, themes, and types of evidence available), link to the review questions and objectives, and consider the relevance to key groups.	15–16
Limitations	20	Discuss the limitations of the scoping review process.	15 (Section 4.5)
Conclusions	21	Provide a general interpretation of the results with respect to the review questions and objectives, as well as potential implications and/or next steps.	17 (Section 5)
**FUNDING**			
Funding	22	Describe sources of funding for the included sources of evidence, as well as sources of funding for the scoping review. Describe the role of the funders of the scoping review.	18 (This research received no external funding.)

JBI = Joanna Briggs Institute; PRISMA-ScR = Preferred Reporting Items for Systematic reviews and Meta-Analyses extension for Scoping Reviews.

* Where *sources of evidence* (see second footnote) are compiled from, such as bibliographic databases, social media platforms, and Web sites.

† A more inclusive/heterogeneous term used to account for the different types of evidence or data sources (e.g., quantitative and/or qualitative research, expert opinion, and policy documents) that may be eligible in a scoping review as opposed to only studies. This is not to be confused with *information sources* (see first footnote).

‡ The frameworks by Arksey and O'Malley (6) and Levac and colleagues (7) and the JBI guidance (4, 5) refer to the process of data extraction in a scoping review as data charting.

§ The process of systematically examining research evidence to assess its validity, results, and relevance before using it to inform a decision. This term is used for items 12 and 19 instead of "risk of bias" (which is more applicable to systematic reviews of interventions) to include and acknowledge the various sources of evidence that may be used in a scoping review (e.g., quantitative and/or qualitative research, expert opinion, and policy document).

From: Tricco AC, Lillie E, Zarin W, O'Brien KK, Colquhoun H, Levac D, et al. PRISMA Extension for Scoping Reviews (PRISMAScR): Checklist and Explanation. Ann Intern Med. 2018;169:467–473. doi: 10.7326/M18-0850.
